# Changes in the chemical composition and medicinal effects of black ginseng during processing

**DOI:** 10.3389/fphar.2024.1425794

**Published:** 2024-11-11

**Authors:** Ye Qiu, Nengyuan Wang, Zhe Yu, Xiao Guo, Ming Yang

**Affiliations:** ^1^ Jiangxi University of Chinese Medicine, Nanchang, Jiangxi, China; ^2^ National Pharmaceutical Engineering Centre for Solid Preparation in Chinese Herbal Medicine, Nanchang, Jiangxi, China; ^3^ Department of Traditional Chinese Medicine, College of Pharmacy, Changchun University of Chinese Medicine, Changchun, China; ^4^ Jilin Cancer Hospital, Changchun, China

**Keywords:** black ginseng, ginsenoside, amino acids, Maillard reaction, transcriptome

## Abstract

**Aim of the Study:**

To study the changes in the chemical composition and medicinal effects of black ginseng during processing.

**Materials and Methods:**

The contents of ginsenosides Rg1, Re, Rh1, Rb1, 20-(S)-Rg3, 20-(R)-Rg3, and Rg5 were determined using high-performance liquid chromatography (HPLC), and the percentage of rare saponins was calculated. Furthermore, changes in the contents of reducing sugars and amino acids (i.e., Maillard reaction (MR) substrates) were measured to assess the relationship between processing and the MR. Compounds were identified using HPLC-MS and their cleavage patterns were analyzed. Gene co-expression network bioinformatics techniques were applied to identify the pharmacological mechanism of black ginseng.

**Results:**

The changes in the physicochemical characteristics of black ginseng during processing were determined based on the MR. Rare saponins accumulated during black ginseng processing. In addition, reducing sugars were produced through polysaccharide pyrolysis and the MR; thus, their content initially increased and then decreased. The amino acid content gradually decreased as the number of evaporation steps increased, indicating that both amino acids and reducing sugars acted as substrates for the MR during black ginseng processing. Thirty-one saponins, 18 sugars, and 58 amino acids were identified based on the MS analysis. Transcriptomics results demonstrated that black ginseng can regulate signaling pathways such as the TNF, IL-17, MAPK, and PI3K-Akt pathways. This finding helps us understand the observed proliferation and differentiation of immune-related cells and positively regulated cell adhesion.

## 1 Introduction

Black ginseng is a black or dark brown product made by repeatedly steaming and drying ginseng (*Panax ginseng* CA Mey.), a plant from the Wugangaceae family. Black ginseng is a fairly new concoction rich in rare saponins. Ginsenosides are the main active substances in black ginseng and exhibit various pharmacological effects, such as anti-tumor (Yang D. et al., 2020; Qing et al., 2020; Zhang et al., 2019; Zheng et al., 2019), anti-oxidant (Choudhry et al., 2019; Hussain et al., 2021; Li et al., 2020; Wu et al., 2023; Wang et al., 2019), anti-inflammatory (Kim et al., 2020; Choi et al., 2021; Xu et al., 2022; Chen et al., 2021), and anti-viral (Kim et al., 2019; Zhang et al., 2023) properties. The appearance of black ginseng powder, tablets, and other products in the market indicates the potential of black ginseng as a dual-purpose product (i.e., as food and medicine) (Heinrich et al., 2022; Chen, 2023; Yang S-h et al., 2023). Therefore, research on the chemical composition and pharmacological effects of black ginseng is crucial.

Black ginseng processing involves the Maillard reaction (MR) (i.e., non-enzymatic browning), one of the most important chemical reactions that occur during food processing and storage. The MR affects food quality. First reported by French scientist L.C. Maillard in 1921, the MR involves a dehydration condensation between carbonyl-containing reducing sugars, aldehydes, ketones, and nitrogenous substances such as proteins, amino acids, and peptides with free amino groups, ultimately producing melanin-like compounds (van Boekel, 2006).

In this paper, we analyzed the changes in chemical composition during black ginseng processing, based on the MR. We investigated the compositional differences between the ginsenosides, reducing sugars, and amino acids in black ginseng and fresh ginseng, using high-performance liquid chromatography-mass spectrometry (HPLC-MS). We also used transcriptomics with gene chip technology to enrich the genes before and after processing, analyzed the differences in biological pathways, and examined changes in pharmacodynamics. This research aims to identify the key points in black ginseng preparation and provide a foundation for the in-depth study of the product’s potential mechanisms. Moreover, we conducted a preliminary study on the differences in the pharmacological mechanisms of black ginseng and fresh ginseng, aiming to offer ideas for further exploration of the underlying mechanisms.

## 2 Materials and methods

### 2.1 Plant materials and reagents

Ginsenosides and glucose with purity greater than 98% were purchased from Shanghai Yuanye Biotechnology Co. Methanol and acetonitrile for chromatography were provided by Thermo Fisher Scientific (Waltham MA, United States). Other chemicals were of reagent grade.

All ginseng samples were purchased from Tonghua Tahuaxing Native Products Co. and certified according to the standards recorded in the Chinese Pharmacopoeia. The first process worked as follow: streaming samples for 1.5 h under atmospheric pressure and heating at 70°C for 18 h. After that, samples were steamed for 2 h, and baked at 60°C for 9 h,which was repeated for nine times.

### 2.2 Preparation of black ginseng extract

Black ginseng (0.2 g) was transferred to a test tube and 30 mL of 40% ethanol aqueous solution was added and mixed well. The sample was placed in a constant-temperature water bath at 80°C for 60 min. Then, the sample was centrifuged at 4,000 r/min for 15 min, and the supernatant was collected and stored at room temperature.

### 2.3 Measurement items and test methods

#### 2.3.1 Determination of ginsenoside content

Ginseng was powdered and sieved four times. Next, 1 g of powder was placed in a Soxhlet extractor, trichloromethane was added, and the mixture was heated by reflux for 3 h. The solvent was then evaporated to dryness. The residue was dissolved with 50 mL of water saturated with n-butanol and passed through filter paper into a 100 mL conical flask, which was tightly plugged and placed overnight under ultrasonication (250 W, 50 kHz). After filtration, 25 mL of filtrate was measured and evaporated. Methanol was added to dissolve the residue, which was transferred into a 5 mL measuring flask.

#### 2.3.2 Determination of reducing sugar content

Glucose (100 mg) was heated in an oven at 80°C to a constant weight and dissolved with water to obtain 1.0 mg/mL and 0.1 mg/mL glucose standard solutions.

To obtain the reducing sugar standard curve, 0, 0.2, 0.4, 0.6, 0.8, 1.0, and 1.2 mL of glucose standard solutions (1 mg/mL) were added to respective test tubes. Distilled water (2 mL) was added and mixed well. Then, 1.5 mL of 3,5-dinitrosalicylic acid solution was added and mixed well. The solutions were heated in a boiling-water bath for 5 min. The volume was adjusted to 10 mL with distilled water and the tubes were removed and cooled down immediately. Absorbance was measured at 520 nm, using an UV spectrophotometer. Readings were taken three times in parallel.

The black ginseng extract (1 mL, obtained as described in Section 2.2) was transferred into a test tube and its absorbance was determined. The content of reducing sugar in the sample was obtained using the standard curve.

#### 2.3.3 Determination of amino acid content

The sample (0.1 g) was mixed with 1 mL of extraction solution at room temperature and centrifuged at 12,000 rpm for 10 min. The supernatant was collected and reagents were sequentially added according to Table 1.

**TABLE 1:** Amino acid content determination.

Reagent (μL)	Measuring tube	Blank tube
Blank tube		40
Supernatant	40	
Chemical reaction max	560	560
Reagent III	40	40
The reagents were mixed in tubes, which were then covered tightly with sealing film to prevent moisture loss and placed in a boiling-water bath for 15 min. Subsequently, the tubes were cooled down to room temperature and shaken for 1 min		
95% ethoxide	320	320
The sample was mixed well; 200 μL of clarified liquid was taken (centrifuged at 8,000 rpm for 5 min at room temperature if turbidity was present) and poured into a 96-well plate. Absorbance (value A) was read at 570 nm		

### 2.4 High-performance liquid chromatography-mass spectrometry (HPLC-MS)

For HPLC, the mobile phase was 0.1% formic acid aqueous solution (A) and acetonitrile (B). The elution gradient was as follows: 0–2 min, 5% B; 2–6 min, 5%–30% B; 6–7 min, 30% B; 7–12 min, 30%–78% B; 12–14 min, 78% B; 14–17 min, 78%–95% B; 17–20 min, 95% B; 20–21 min, 95%–5% B; 21–25 min, 5% B. The column temperature was 30°C, the flow rate was 0.3 mL/min, the injection volume was 2 mL/min, and the autosampler temperature was 4°C.

For MS, the positive and negative ion modes were used. In both cases, the source was kept at 325°C. The sheath gas, auxiliary gas, and purging gas flow rates were 45, 15, and 1 arb, respectively. The electrospray voltage was 3.5 kV, the capillary temperature was 330°C, and the S-Lens RF level was 55%.

The scanning mode was full scan (*m/z* 100∼1,500) with data-dependent secondary mass spectrometry (dd-MS2, TopN = 5) and resolution of 120,000 (primary mass spectrometry) and 60,000 (secondary mass spectrometry). The collision mode was high-energy collisional dissociation (HCD).

### 2.5 Transcriptomics testing

The black ginseng aqueous fraction was used as the positive control. The human breast cancer cell line MCF-7 served as the model. The IC_50_ concentration of the screening drug was 5 mg/mL. MCF-7 cells were inoculated in 96-well plates at 1×10^4^ cells/100 μL, and cultured for 24 h. Subsequently, the black ginseng aqueous fraction was added to the cells at three concentrations (5, 2.5, and 1.25 mg/mL). Black ginseng aqueous fraction alone was used as the negative control. Three-year-old aqueous extracts served as the negative control groups. After an additional 24 h of incubation, total RNA was extracted. Transcriptome changes were detected using a high-efficiency, high-throughput gene detection system (HISTAG), with three biological replicates per concentration.

## 3 Results

### 3.1 Chemical composition of ginseng and black ginseng

#### 3.1.1 Changes in the ginsenoside content

**FIGURE 1:**
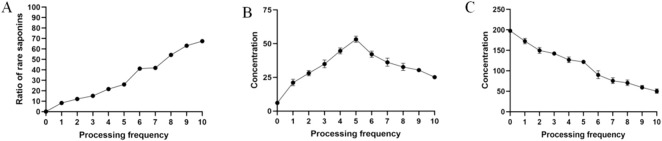
Changes in the chemical composition. **(A)** Changes in rare saponins; **(B)** changes in the reducing sugar content; **(C)** changes in the amino acid content.

Ginsenosides Rg1, Re, Rh1, Rb1, 20-(S)-Rg3, 20-(R)-Rg3, and Rg5 were used as indicators to assess the MR’s progress by calculating the proportion of rare saponins within the total saponin content. The results (Figure 1A) indicate a steady increase in the proportion of rare saponins under atmospheric pressure. The conversion of ginsenosides was particularly high during the sixth steaming process, after which it slowed down again. This pattern reflects the progression of the MR.

#### 3.1.2 Changes in the reducing sugar content

Reducing sugar, as the MR’s substrate, indicates the progress of the reaction to a certain extent. During ginseng steaming, the reducing sugar content initially increases and then decreases as the number of steaming cycles increases (Figure 1B). This is primarily due to the abundance of polysaccharides in fresh ginseng, which are degraded during high-temperature processing. In the reaction’s early stages, the degradation rate of these polysaccharides is faster, increasing the reducing sugar content. However, as the steaming cycles continue, the original polysaccharides in ginseng are depleted, and reducing sugar production slows down. Because reducing sugars continually react with amino compounds during the MR, their contents reach a plateau and remain stable until the end of the process.

#### 3.1.3 Changes in the amino acid content

The basis of the MR is the condensation of amino compounds with carbonyl compounds (reducing sugars). Amino acids, as substrates of the MR, indicate the reaction’s progression. The amino acid content decreased steadily during the first five evaporation cycles under atmospheric pressure (Figure 1C). The most rapid decrease was observed during the sixth evaporation. This may be explained by the reducing sugar content peaking after the fifth evaporation, leading to a higher substrate concentration and an accelerated reaction between the carbonyl and amino groups. Afterward, the decrease in the amino acid content slowed down again until the end of the steaming process. Thus, we confirmed that both amino acids and reducing sugars are substrates in the MR.

### 3.2 Results of mass spectrometry

**FIGURE 2:**
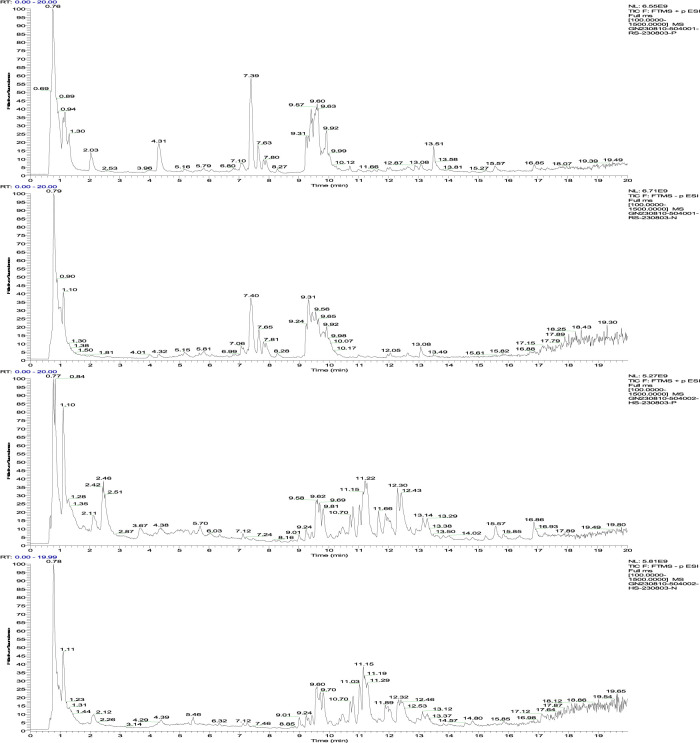
**(A)** Positive ion flow diagram of fresh ginseng; **(B)** negative ion flow diagram of fresh ginseng; **(C)** positive ion flow diagram of black ginseng; **(D)** negative ion flow diagram of black ginseng.

The HPLC-MS analysis was conducted in both the positive and negative ion modes. The resulting total ion chromatograms are displayed in Figure 2. These chromatograms reveal notable differences in the chemical composition of fresh and black ginseng, indicating that repeated steaming of fresh ginseng affects its chemical composition.

#### 3.2.1 Ginsenoside mass spectrometry results

Ginsenosides can be classified into dammarane-type, ocotillo-type, and oleanolic acid-type ginsenosides based on their glycoside structures. Most dammarane-type ginsenosides are triterpenoids and can be further categorized as protoginseng diols (PPDs) and protoginseng triols (PPTs). We used Compound Discoverer 3.3 to compare the MS of fresh ginseng with that of black ginseng (i.e., processed by high-temperature steaming). These samples were classified and analyzed to identify ginsenosides, using secondary MS data from the mzCloud and mzVault databases (Thermo Fisher Scientific). The results revealed substantial differences between the ginsenoside profiles of fresh ginseng and black ginseng. Thirty-one ginsenosides were identified: 16 from fresh ginseng and 26 from black ginseng, with 11 common saponins, most of which decreased during processing (Table 2).

**TABLE 2:** Ginsenoside mass spectral data.

Number	RT [min]	Name	Annot. DeltaMass [ppm]	Annotation MW	Calc. MW	Ginsenoside type	Ginseng	Number	Trend of change
1	5.433	Pseudoginsenoside F11	0.04	800.49	800.49	Ocotillon	+		
2	6.025	Ginsenoside F1	0.38	638.44	638.44	PPT	+	+	↑
3	7.362	Ginsenoside Rg1	0.94	946.55	946.55	PPT	+	+	↓
4	7.400	20-O-Glucoginsenoside Rf	−0.62	800.49	800.49	PPT	+	+	↓
5	8.645	Ginsenoside Rf	1.14	962.55	962.55	PPT	+		
6	9.238	Ginsenoside Rb1	−0.71	800.49	800.49	PPT	+	+	↓
7	9.316	Ginsenoside Rb2	−1.36	1108.60	1108.60	PPD	+		
8	9.439	Ginsenoside Rb3	−0.29	1078.59	1078.59	PPD	+		
9	9.443	Ginsenoside F3	−1.13	1078.59	1078.59	PPD	+	+	↓
10	9.549	Ginsenoside Rh3	0.87	770.48	770.48	PPT	+	+	↓
11	9.556	Ginsenoside Rg2	−1.24	604.43	604.43	PPD	+	+	↑
12	9.581	Ginsenoside Rb1	0.81	784.50	784.50	PPT	+	+	↓
13	9.645	20-R-Glucoginsenoside Rh1	−0.72	1108.60	1108.60	PPD		+	
14	9.693	Ginsenoside Rc	−0.85	638.44	638.44	PPT		+	
15	9.715	Ginsenoside Rd	−0.24	1078.59	1078.59	PPD	+	+	↓
16	9.715	Ginsenoside	0.45	1078.59	1078.59	PPD	+		
17	9.716	20-S-Glucoginsenoside Rg3	−1.00	784.50	784.50	PPD		+	
18	9.716	20(R)-Protopanaxadiol	−1.39	460.39	460.39	PPD		+	
19	9.798	20-S-Glucoginsenoside Rh1	1.42	638.44	638.44	PPT		+	
20	10.242	20-O-Glucoginsenoside Rf	1.04	962.55	962.55	PPT		+	
21	10.482	Ginsenoside compound K	−0.92	622.44	622.44	PPD		+	
22	10.490	20-R-Glucoginsenoside Rg3	−0.98	784.50	784.50	PPD		+	
23	10.702	Ginsenoside Rk1	−1.12	766.49	766.49	PPD		+	
24	10.799	Ginsenoside Rg5	1.37	766.49	766.49	PPD		+	
25	10.990	Oleanolic acid	−1.14	456.36	456.36	Oleanolane	+	+	↑
26	11.029	Ginsenoside Rh4	−0.53	620.43	620.43	PPT		+	
27	11.109	Panaxytriol	−1.57	476.39	476.39	PPT		+	
28	11.145	Ginsenoside Rk3	1.05	620.43	620.43	PPT		+	
29	11.220	Ginsenoside F2	1.65	784.50	784.50	PPD	+	+	↑
30	11.891	Pseudoginsenoside F11	0.32	826.51	826.51	PPT		+	
31	12.567	Ginsenoside F1	0.64	826.51	826.51	PPT		+	

The results showed that the ginsenosides’ types and contents changed notably after repeated steaming. The contents of the original ginsenosides (e.g., Rg1, Re, Rb1, Rb2, Rb3, and Rc) decreased while rare saponins, such as ginsenosides 20-(R)-Rg3, 20-(S)-Rg3, Rk1, Rk3, and Rg5 appeared. The MS ion fragmentation patterns together with the ginsenoside structures helped us infer the transformation process of ginsenosides, described below.

**FIGURE 3:**
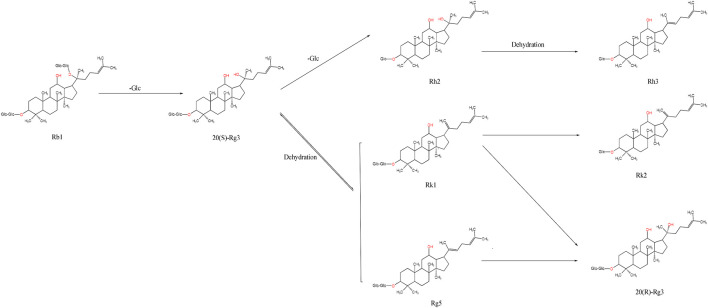
Schematic diagram of the transformation of ginsenoside Rb1.

Ginsenosides such as Rb1, Rb2, Rb3, CK, and Rc are PPDs. The presence of hydroxyl or hydroxyl substitutions at the C-3 and C-20 positions is a common structural feature of these ginsenosides. During high-temperature processing, glycosyl groups at the C-3 and C-20 positions of these ginsenosides are hydrolyzed and replaced with hydroxyl groups of varying molecular weights. For instance, when ginsenoside Rb1 is heated, the disaccharide group at the C-20 position is hydrolyzed and replaced with a hydroxyl group, generating ginsenoside 20-(S)-Rg3. This product can undergo further hydrolysis to remove a glucose molecule at C-3, resulting in ginsenoside Rh2. Dehydroxylation at C-20 releases a water molecule, converting Rh2 into ginsenoside Rh3. The hydroxyl group at C-20 of 20-(S)-Rg3 is unstable and dehydrates between C-20 and C-21 to generate ginsenoside Rk1. If dehydration occurs between C-20 and C-22, ginsenoside Rg5 is formed. Moreover, the unsaturated double bond positions in ginsenosides Rk1 and Rg5 can undergo an addition reaction, producing ginsenoside 20-(R)-Rg3 (Figure 3). These substituents can be hydrolyzed and replaced by hydroxyl groups during high-temperature processing because of the structural feature of diol-type saponins, which mainly differ by the substituents at C-20, generating the corresponding rare saponins. Therefore, the contents of 20-(S)-Rg3, 20-(R)-Rg3, Rk1, and Rg5 in black ginseng increase. In addition, the glucose molecule at C-3 of ginsenoside Rk1 can be removed to generate ginsenoside Rk2.

**FIGURE 4:**
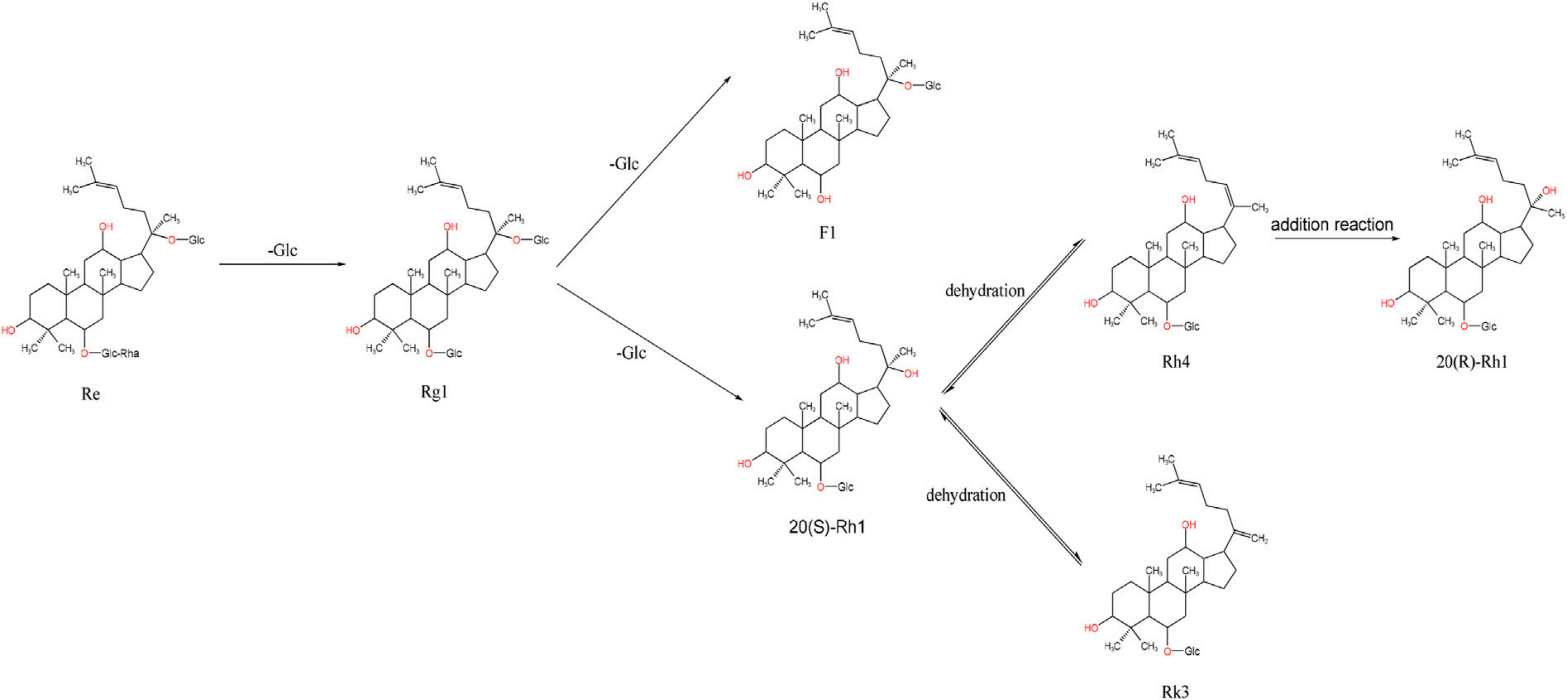
Schematic diagram of the transformation of ginsenosides Re and Rg1.

Ginsenosides such as Re, Rg1, Rg2, and Rf are PPTs, based on PPDs with the addition of hydroxyl or hydroxyl substitutions at the C-6 position. For instance, ginsenoside Re can be converted into Rg1 by removing the rhamnoside (Rha) at the C-6 position. During steaming, Rg1 undergoes hydrolysis at the C-6 position, where the original sugar group is replaced with a hydroxyl group, producing ginsenoside F1. If F1 loses the sugar group at the C-20 position, it forms 20-(S)-Rh1. Further dehydration of 20-(S)-Rh1 creates an unsaturated bond at the C-20 or C-22 positions, resulting in ginsenoside Rk3. If a water molecule is removed between the C-20 and C-21 positions, another unsaturated bond forms, generating Rh4. Finally, ginsenoside Rh4 can undergo addition at the C-20 or C-21 positions to form 20-(R)-Rg3 (Figure 4).

In summary, the original saponins in ginseng are transformed during high-temperature processing, which can trigger a series of chemical reactions, such as deglycosylation, dehydration, and addition, producing rare saponins unique to black ginseng, such as the ginsenosides 20-(S)-Rg3, 20-(R)-Rg3, and Rk1. When ginseng is steamed, the sugar groups are reduced, enabling these, the hydroxyl groups, and other polar groups to form complexes with cholesterol in the cell membranes. This interaction alters the permeability and function of the cell membranes, influencing ginseng’s pharmacological effects. For instance, saponins from fresh ginseng cannot effectively regulate serum corticosterone and adrenocorticotropic hormone (ACTH) levels. By contrast, deglycosylated derivatives from black ginseng, such as Rg3 and CK, increase ACTH levels and normalize the hyperactivity of the HPA axis (Zhang et al., 2016), resulting in antidepressant effects. However, Rh2 lacks this effect, which may be due to the position of the sugar substituent, a hypothesis that requires further verification. Furthermore, black ginseng obtained through steaming (but not fresh ginseng) exhibits strong memory-enhancing effects. This effect was attributed to secondary saponins like Rg5 and Rg3 (Lee et al., 2020).

#### 3.2.2 Sugar and amino acid mass spectrometry results

The transformation of sugars and amino acids in black ginseng was investigated. Black ginseng was prepared by steaming fresh ginseng at 117°C for 1.5 h, followed by drying at 68°C; this cycle was repeated three times. The results revealed that the sugars and amino acids in black ginseng were markedly different from those in fresh ginseng (Tables 3, 4). Eighteen sugars were detected, seven in the positive ion mode and 11 in the negative ion mode. Of these, eight compounds were found in fresh ginseng and 10 in black ginseng. Furthermore, 58 amino acids were detected, 45 in the positive ion mode and 13 in the negative ion mode. Among them, 49 compounds were detected in fresh ginseng and nine in black ginseng.

**TABLE 3:** Sugars detected by HPLC-MS.

Number	RT [min]	Name	Annot. DeltaMass [ppm]	Annotation MW	Calc. MW	Ionic mode	Ginseng	Black ginseng
1	0.771	Hex-2-ulose	−1.06	180.06339	180.0632	+		+
2	0.771	Galactinol	−1.29	342.11621	342.11577	+		+
3	0.773	D-(+)-Maltose	−0.29	342.11621	342.11611	−		+
4	0.777	D-Sucrose	−1.1	342.11621	342.11583	+	+	
5	0.784	Raffinose	0.27	504.16903	504.16917	−		+
6	0.793	α,α-Trehalose	−0.33	342.11621	342.1161	−	+	
7	0.815	Trehalose-6-phosphate	0.23	422.08254	422.08264	−		+
8	0.825	D-Glucono-1,5-lactone	−4.67	178.04774	178.04691	−		+
9	0.85	1,4-D-xylobiose	−0.56	282.09508	282.09492	−		+
10	1.075	Nystose	1.21	666.22186	666.22267	−	+	
11	2.131	Glucosylisomaltol	−1.66	288.08452	288.08404	+		+
12	4.858	1-(4-Hydroxybenzoyl)glucose	−0.51	300.08452	300.08436	−	+	
13	5.038	Caffeic acid hexoside	−0.38	342.09508	342.09495	−		+
14	7.072	Polydextrose	−1.54	283.13208	283.13164	+	+	
15	7.145	Sibiricose A1	0.27	548.17412	548.17427	−	+	
16	9.313	α-Lactose	−1.83	342.11621	342.11558	+	+	
17	9.315	Maltotetraose	−1.16	666.22186	666.22109	+	+	
18	17.313	3-O-Undecyl-D-glucopyranose	1.02	334.23554	334.23588	−		+

**TABLE 4:** Amino acids detected by HPLC-MS.

Number	RT [min]	Name	Annot. DeltaMass [ppm]	Annotation MW	Calc. MW	Ionic mode	Ginseng	Black ginseng
1	0.684	D-(+)-Pipecolinic acid	−0.28	129.08	129.08	+	+	
2	0.695	L-Histidine	0.41	155.07	155.07	+	+	
3	0.743	N-Methylthreonine	−3.56	133.07	133.07	−	+	
4	0.761	DL-Arginine	−0.25	174.11	174.11	+	+	
5	0.799	D-(+)-Proline	1.83	115.06	115.06	+	+	
6	0.834	Acetylarginine	0.64	216.12	216.12	+	+	
7	0.910	N-Acetylhistamine	−0.31	153.09	153.09	+	+	
8	1.067	Valine	1.52	117.08	117.08	+	+	
9	1.094	L-(−)-Methionine	0.06	149.05	149.05	+	+	
10	1.118	N-Fructosyl tyrosine	−0.51	343.13	343.13	+	+	
11	1.194	L-Tyrosine	−4.87	181.07	181.07	−	+	
12	1.207	L-Isoleucine	0.3	131.09	131.09	+	+	
13	1.265	N-Fructosyl isoleucine	−1.26	293.15	293.15	+	+	
14	1.300	L-Norleucine	0.3	131.09	131.09	+	+	
15	1.309	Prolylleucine	−0.56	228.15	228.15	+	+	
16	1.404	Leucylalanine	−0.21	202.13	202.13	+	+	
17	1.443	L-Threo-3-phenylserine	−0.39	181.07	181.07	+		+
18	1.485	Valylproline	−0.27	214.13	214.13	+	+	
19	1.580	Valylvaline	−0.17	216.15	216.15	+	+	
20	1.791	Acetylproline	−0.25	157.07	157.07	+	+	
21	2.229	5-Hydroxytryptophan	−0.2	220.08	220.08	+	+	
22	2.302	Serylleucine	−0.24	218.13	218.13	+	+	
23	2.431	γ-Glutamyltyrosine	−0.83	310.12	310.12	+	+	
24	2.564	Metyrosine	−0.41	195.09	195.09	+		+
25	2.612	Ala-Ile	0.02	202.13	202.13	+	+	
26	2.629	Ala-Leu	−3.62	202.13	202.13	−	+	
27	2.637	L-Phenylalanine	0.2	165.08	165.08	+	+	
28	2.638	Threonylleucine (isomer of 809, 926)	−0.27	232.14	232.14	+	+	
29	2.687	Glycyl-L-leucine	−0.09	188.12	188.12	+	+	
30	3.757	H-LEU-VAL-OH	−0.2	230.16	230.16	+	+	
31	4.243	Ala-Phe	−0.98	236.12	236.12	+	+	
32	4.254	Leucylproline	−0.9	228.15	228.15	+	+	
33	4.305	DL-Tryptophan	−0.19	204.09	204.09	+	+	
34	4.319	D-(+)-Tryptophan	−3.08	204.09	204.09	−	+	
35	4.421	Leucylvaline	−0.8	230.16	230.16	+	+	
36	4.521	γ-Glutamylleucine	−0.47	260.14	260.14	−	+	
37	4.552	N-Acetylhomoproline	−0.82	171.09	171.09	+		+
38	4.664	N-Acetyl-L-tyrosine	−2.2	223.08	223.08	−	+	
39	5.037	Cyclo (Pro-Val)	−0.57	196.12	196.12	+		+
40	5.040	Glutamylphenylalanine (isomer of 1,503)	−0.63	294.12	294.12	+	+	
41	5.250	H-PHE-VAL-OH	−0.95	264.15	264.15	+	+	
42	5.571	Leucylleucine	−0.67	244.18	244.18	+	+	
43	5.576	Isoleucylisoleucine	−1.24	244.18	244.18	−	+	
44	5.696	N-Benzoyl-DL-alanine	−0.66	193.07	193.07	+		+
45	6.121	Phenylalanylisoleucine (isomer of 1,329)	−0.88	278.16	278.16	+	+	
46	6.161	N-Acetyl-L-leucine	−0.07	173.11	173.11	+	+	
47	6.166	2-(Acetylamino)hexanoic acid	−4.94	173.11	173.10	−		+
48	6.266	Glu-Val-Phe	−0.84	393.19	393.19	+	+	
49	6.359	Cyclo (leucyl-prolyl)	−1.02	210.14	210.14	+		+
50	6.680	N-Acetylphenylalanine	−0.38	207.09	207.09	+	+	
51	6.871	Malonyltryptophan	−1.72	290.09	290.09	+	+	
52	6.872	N-Acetyl-DL-tryptophan	−0.91	246.10	246.10	−	+	
53	6.923	2-Hydroxyphenylalanine	−0.62	181.07	181.07	+		+
54	7.911	L-Methionyl-L-alpha-aspartyl-L-valyl-L-asparaginyl-L-prolyl-L-phenylalanyl-L-leucyl-L-leucyl-L-phenylalanyl-L-leucyl-L-lysyl-L-valyl-L-prolyl-L-isoleucyl-L-glutamine	−1.17	1772.99	1772.99	−	+	
55	9.388	N-Acetyl-L-phenylalanyl-L-glutaminyl-L-seryl-L-cysteinylglycyl-L-asparaginyl-L-valyl-L-phenylalanyl-L-valyl-L-alpha-aspartylglycyl-L-tyrosyl-L-phenylalanyl-L-alpha-glutamyl-L-arginyl-L-leucyl-L-arginy l-L-alanyl-L-lysyl-L-leucinamide	3.26	2389.20	2389.21	−	+	
56	9.558	L-Tyrosyl-L-seryl-L-phenylalanyl-L-lysyl-L-alpha-aspartyl-L-alanyl-L-prolyl-L-leucyl-D-alanyl-N∼5∼-(diaminomethylene)-L-ornithine	0.02	1166.61	1166.61	−	+	
57	9.786	Glycyl-L-seryl-O-octanoyl-L-seryl-L-phenylalanyl-L-leucyl-L-seryl-D-prolyl-L-alpha-glutamyl-L-histidyl-L-glutamamide	0.63	1212.61	1212.61	−	+	
58	10.066	3-(2-Naphthyl)-L-alanine	−1.19	215.09	215.09	+		+

The results showed that the sugar and amino acids in black ginseng differed from those in fresh ginseng. In the latter, oligosaccharides and polysaccharides are the predominant sugars, and peptides and single amino acids are the predominant amino compounds. During high-temperature processing, the polysaccharides in ginseng are hydrolyzed to produce monosaccharides, and the peptide chains between the amino acids are broken. Simultaneously, the amino groups of amino acids combine with the carbonyl groups of ketose sugars, generating monosaccharide and glucose derivatives. Thus, the sugars in black ginseng are mainly monosaccharides and glucose derivatives, while the amino acids are predominantly acetylated or phenylated derivatives. This result further confirms that black ginseng processing involves the MR.

### 3.3 Differential gene identification

Three concentrations (5, 2.5, and 1.25 mg/mL) of three-year-old black ginseng aqueous extract were used as controls. MCF-7 cells treated with these three concentrations served as the experimental group. The FC-t algorithm was used to identify differentially expressed genes. The thresholds were set at *P* < 0.05, and |FC|>1.5, allowing for the identification of differential genes across the three groups at different concentrations.

**FIGURE 5:**
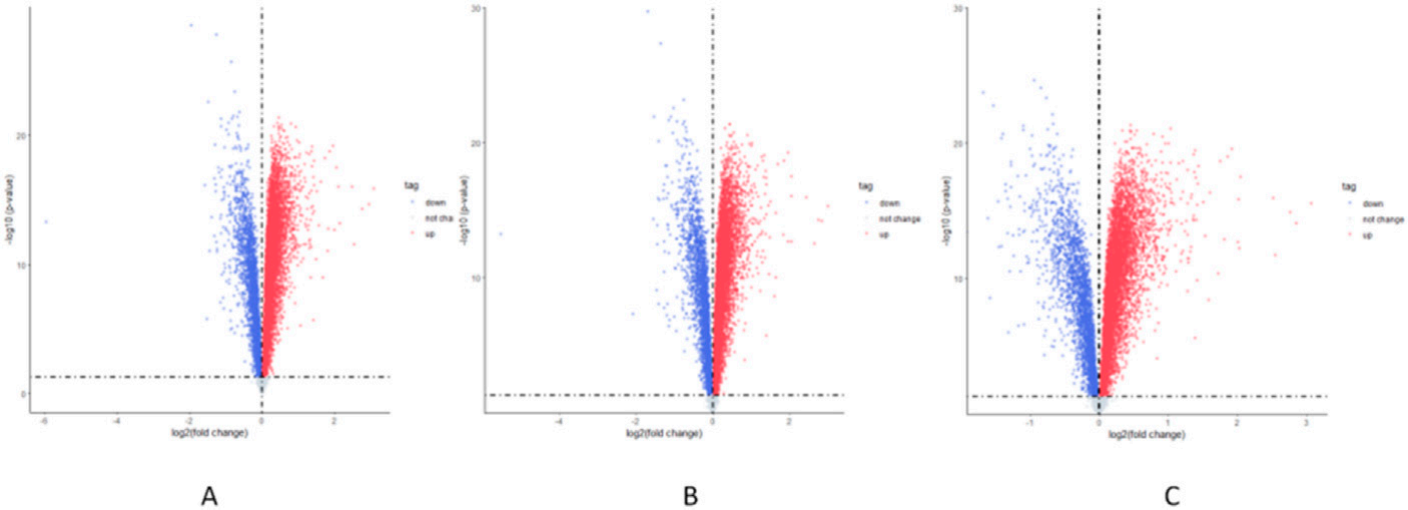
Results of differential gene analysis. **(A)** Cells exposed to the low-concentration extract; **(B)** cells exposed to the medium-concentration extract; **(C)** cells exposed to the high-concentration extract. The blue color indicates a decrease in expression, whereas red indicates an increase in expression.

The differentially expressed genes before and after administering the black ginseng extract were identified: 683 genes from the cells exposed to the low-concentration extract, 668 genes from the cells exposed to the medium-concentration extract, 682 genes from the cells exposed to the high-concentration extract, and 879 genes when combining the data from all three concentrations (Figure 5).

#### 3.3.1 Gene co-expression network construction and gene module delineation

Gene co-expression networks are commonly applied in transcriptomics. In this study, data from 879 genes—derived from the differential genes detected in the cells exposed to the three extract concentrations—were selected as background data. The Pearson correlation algorithm was used to construct the relationship matrix between the differential genes. Relationships with |r| > 0.7 and *P* < 0.05 were screened to construct the gene co-expression network.

The multilevel algorithm was used to divide the gene modules. Modules with few genes were removed while those with more genes were analyzed. As a result, seven gene modules were obtained. Table 5 shows the number of genes in each module.

**TABLE 5:** Number of genes in the modules.

Module number	Number of genes
1	132
2	106
3	83
4	110
5	184
6	154
7	139

#### 3.3.2 GO and KEGG analysis of each gene module

**FIGURE 6:**
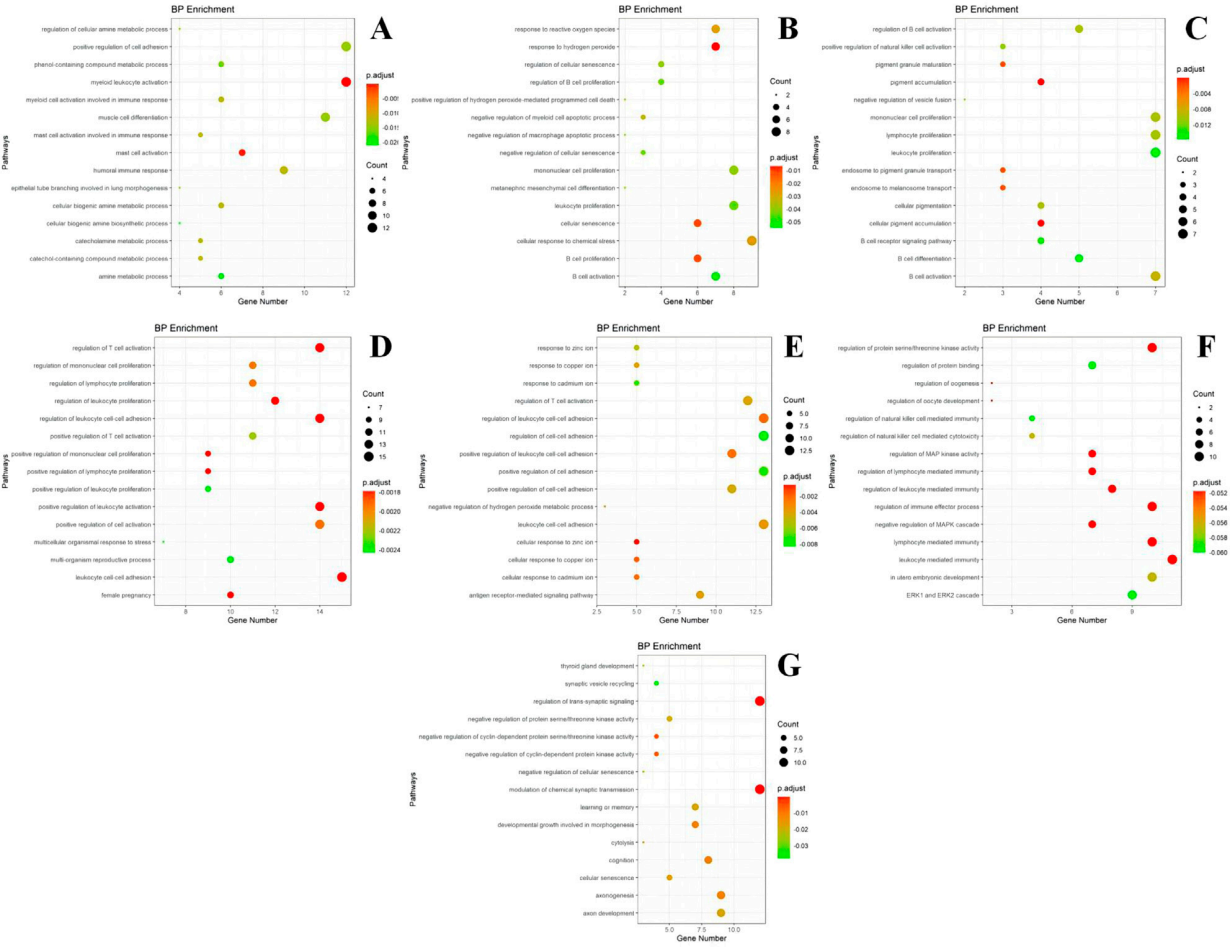
GO enrichment results of different modules. **(A)** module m1 GO enrichment analysis results; **(B)** module m2 GO enrichment analysis results; **(C)** module m3 GO enrichment analysis results; **(D)** module m4 GO enrichment analysis results; **(E)** module m5 GO enrichment analysis results; **(F)** module m6 GO enrichment analysis results; **(G)** module m7 GO enrichment analysis results. The size of the dots represents the number of genes in the entry (count value), and the color of the dots represents the degree of enrichment (*P*-value) in a gradient from yellow to red; a redder color indicates a higher degree of enrichment.

**FIGURE 7:**
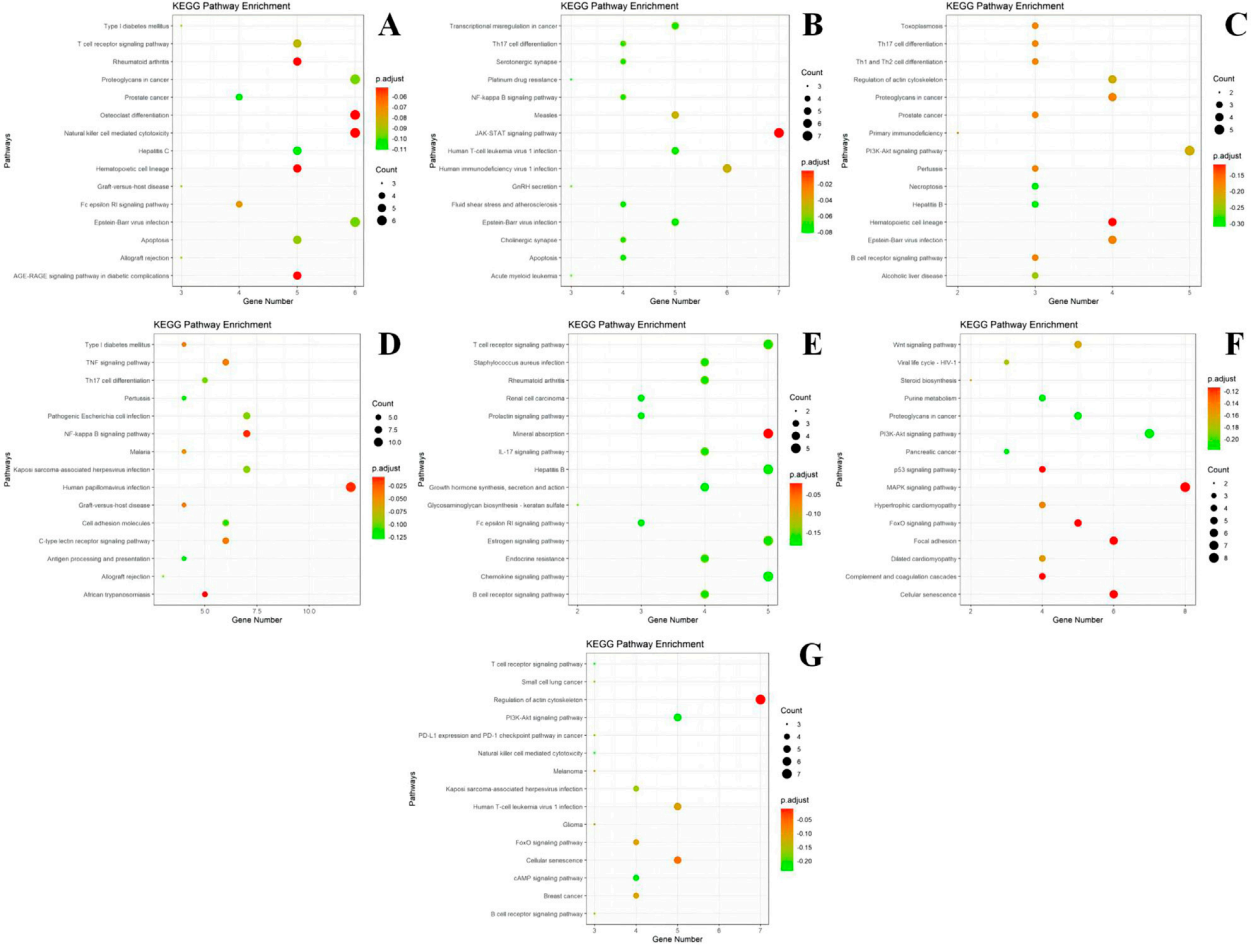
KEGG enrichment results of different modules. **(A)** module m1 KEGG enrichment analysis results; **(B)** module m2 KEGG enrichment analysis results; **(C)** module m3 KEGG enrichment analysis results; **(D)** module m4 KEGG enrichment analysis results; **(E)** module m5 KEGG enrichment analysis results; **(F)** module m6 KEGG enrichment analysis results; **(G)** module m7 KEGG enrichment analysis results. The size of the dots represents the number of genes in the entry (count value) and the color of the dots represents the degree of enrichment (*P*-value) in a gradient from yellow to red; a redder color indicates a higher degree of enrichment.

The genes within each module were enriched and analyzed using biological pathways (BP) and the KEGG (https://www.genome.jp/kegg/) and GO (http://geneontology.org/) databases. The 20 biological processes and KEGG pathways with the smallest *P*-value for each module were selected for visualization. The GO and KEGG enrichment results corresponding to the genes in each module are presented in Figures 6, 7.

Based on the GO enrichment analysis of the differentially expressed genes, most of the enriched entries are BP. Module I is primarily involved in the positive regulation of cell adhesion, skeletal leukocyte activation, myocyte differentiation, humoral immunity, and mast cell activation. Module II is mainly associated with the cellular response to chemical stress, leukocyte proliferation, monocyte proliferation, and response to peroxides and hydrogen peroxide. Module III is primarily involved in monocyte proliferation, lymphocyte proliferation, leukocyte proliferation, and B-cell activation, differentiation, and activation regulation. Module IV participates in leukocyte cell adhesion, promotes leukocyte activation, and regulates leukocyte proliferation. Module V is involved in T-cell activation and responses to zinc and other metal ions such as copper and cadmium. Module VI participates in the regulation of immune effects, mediating the regulation of immunity by leukocytes and lymphocytes. Module VII is involved in the regulation of trans-synaptic signaling, chemosynaptic transmission, and axonal ontogeny and development.

According to the results of KEGG enrichment analysis, most of the genes in Module I are involved in pathways that regulate proteoglycan, skeletal differentiation, natural killer (NK) cell-mediated cytotoxicity in cancer, the AGE-RAGE signaling pathway in diabetic complications, and the T-cell receptor signaling pathway. Module II is implicated in the JAK-STAT signaling pathway, human immunodeficiency virus type 1 (HIV-1) infection, and NF-κB signaling pathway. Module III participates in the PI3K-Akt signaling pathway and Th17, Th1, and Th2 cell differentiation. Module IV is involved in the TNF signaling pathway. Module V is associated with the IL-17 signaling pathway, *Staphylococcus aureus* infection, B-cell receptor signaling pathway, and rheumatoid arthritis. Module VI regulates the MAPK, PI3K-Akt, and Wnt signaling pathways, as well as cellular senescence. Module VII participates in the regulation of the actin cytoskeleton.

#### 3.3.3 Hub gene identification

The PageRank algorithm was used to calculate the importance of each gene in the protein interaction network. The 10 genes with the highest algorithm scores were identified as the module’s hub genes, representing the most critical genes in the network. The results are displayed in Table 6.

**TABLE 6:** Hub genes by module.

1	2	3	4	5	6	7
FRS2	C18ORF1	LOC652493	RPS6KB1	TPST2	QPCT	HBD
INHBB	CD3D	IGKV1D-13	ATP6V1B1	AKAP17A	GNL3L	HBG1/HBG2
PAH	FLRT3	POU2AF1	GALNT3	FOXD1	ITGA8	CUX2
ZGPAT	DNASE1L3	LPHN2	PLIN2	EGR3	WSCD1	GABBR1
MS4A6A	IGHA 2 IGHG3/IGHM	AUTS2	C13ORF15	RHCG	NMU	CRABP2
POLM	BCL2	CD19	IDO1	MTUS1	HIST1H3D	PACSIN2
NNMT	OGDHL	CPLX3/LMAN1L	HIST1H2BH	ACAT2	TGFB1I1	FSCN1
MBD5	RPS5	PDLIM5	SPON1	C1ORF63	PHLDA2	STON1
CXCL9	BCL11A	IGKC/IGKV3-20	ADM	SPDEF	TPM2	MICB
LGALS1	ATF3	LOC732160/NDUFA10	ICAM3	SPARC	CYTH2	NXT1

A literature review identified several hub genes associated with biological processes and conditions. FRS2, FLRT3, RPS5, and PDLIM5 are related to cell differentiation and proliferation. INHBB, ZGPAT, MS4A6A, NNMT, LGALS1, BCL2, BCL11A, TGFB1I1, and FSCN1 are associated with tumors, with BCL2 and BCL11A noted for their anti-apoptotic effects. MBD5, OGDHL, AUTS2, EGR3, QPCT, and ITGA8 are linked to neurological diseases. Moreover, CXCL9, CD3D, DNASE1L3, CD19, ICAM3, and SPDEF are involved in immune regulation and inflammatory responses. Finally, ATF3, LMAN1L, and ACAT2 participate in metabolism.

## 4 Discussion

By analyzing the changes in the reducing sugar, amino acid, and ginsenoside content during black ginseng processing, we can conclude that the MR occurs during repeated steaming. This reaction consumes reducing sugars and amino acids, thereby lowering their contents. The final product of the MR, melanoidin, changes chromaticity (i.e., the color gradually deepens from an initial yellowish-white to dark brown, and, finally, to black). The repeated steaming of black ginseng is accompanied by a decrease in reducing sugars; thus, excessive steaming can result in a sour and astringent product. Therefore, the conversion rate of the active ingredients in black ginseng should be considered, while minimizing the degradation of other nutrients.

Thirty-two saponins, 18 sugars, and 58 amino acids were identified by MS. Of these, 16 saponins were found in fresh ginseng and 26 in black ginseng, including 11 common saponins. Moreover, eight sugars were found in fresh ginseng and 10 in black ginseng. In addition, 49 amino acids were identified in fresh ginseng and nine in black ginseng. These results indicate that ginseng undergoes a series of reactions during high-temperature processing, including hydrolysis, desugarization, and addition. These reactions are based on the instability of the tertiary carbonyl at the C-20 position of dammarane-type saponins, which makes the compounds prone to hydrolysis under high-temperature conditions. This results in the cleavage of the sugar substituent at the C-20 position, the generation of hydroxyl substituents, and further hydrolysis to produce unsaturated saponins.

Ginseng primarily undergoes deglycosylation and dehydration during steaming to produce secondary saponins. The different positions and quantities of the sugar substituents affect ginseng’s pharmacological activities. The anticancer effect of ginsenosides is inversely related to the number of sugar groups they contain (Sun et al., 2011; Ahuja et al., 2018). For example, the original saponins Rc and Rb1 in ginseng have almost no anticancer activity, whereas the secondary saponins Rg3 and Rh2 exhibit anticancer activity (Liu et al., 2024; Wang et al., 2021; Wu et al., 2024; Zhang et al., 2021). Furthermore, the pharmacological effect of Rg3 (two sugar groups) is weaker than that of Rh2 (one sugar group).

There are also notable differences in the pharmacological effects of Rg5 and Rh2. Both can be obtained by removing one sugar group from Rg3. When the sugar group at the C-20 position is removed, Rg5, which exhibits antidepressant efficacy, is generated. By contrast, the Rh2 generated at the C-3 position does not possess such an effect. The MR occurs concurrently during steaming, hydrolyzing polysaccharides and polypeptides to generate small-molecule compounds. The amino compounds in the system can be dehydrated and condensed with the carbonyl compounds (reducing glycosides). High-molecular-weight melanoidins are formed through a series of chemical reactions such as condensation, polymerization, decomposition, and cyclization. This process gradually deepens the color of ginseng and imparts a characteristic aroma. The conversion of secondary saponins during black ginseng processing may be closely related to the MR. Some studies have reported that the products generated by the MR in ginseng have strong free-radical-scavenging activity (Park et al., 2018). However, the structure and formation mechanism of melanoidins remain unclear. Bruhns et al. (2019) suggested that the ratio of carbohydrates to amino acids required for melanoidin synthesis could be inferred from the carbon-to-nitrogen ratio in the final product. Experimental evidence suggests that eliminating water molecules leads to the rearrangement of fragments containing unsaturated portions, resulting in the formation of a chromophore system and, ultimately, melanoidin production, changing the product’s color.

A total of 879 genes were identified by comparing the genetic changes in cells exposed to ginseng extracts before and after steaming at three concentrations, using gene chip technology (Gao et al., 2023). The differential genes identified were used to construct a relationship matrix using the correlation algorithm proposed by Erskine. Relationships with |r| > 0.7 and *P* < 0.05 were selected to construct the gene co-expression network. Subsequently, the multilevel algorithm was applied to divide the gene modules, resulting in seven modules.

Most of the GO enrichment results were related to the BP pathway. Compared to cells exposed to fresh ginseng, the differential genes detected in cells exposed to black ginseng are more effective in regulating cell adhesion and promoting biological pathways such as signaling, cancer metastasis, and immune responses. Furthermore, the gene enrichment results in the fifth module indicate that these genes may be involved in the cellular response to metal ions such as copper, zinc, and cadmium. The cellular response to metal ions influences cellular autophagy, where the cell maintains stability by clearing deformed proteins or senescent dead cells. For example, high zinc ion concentrations promote the formation of autophagic vesicles in alcohol-induced hepatocellular carcinoma cells and are associated with dopamine-induced autophagy in PC12 cells (Hung et al., 2013). Zinc ions also promote autophagosome formation in triamcinolone acetonide-induced breast cancer cells. Similarly, in mouse macrophages, zinc ion deficiency activates caspase-1 to promote apoptosis (Summersgill et al., 2014). Thus, black ginseng powder may enhance the cellular response to metal ions and, thereby, induce cellular autophagy. This could be useful for developing new drugs for neurological diseases, tumors, and related diseases.

The KEGG results revealed that the differential genes identified after ginseng and black ginseng administration were mostly present in the TNF, IL-17, MAPK, and PI3K-Akt signaling pathways, all of which are closely related to inflammation (Liu X. et al., 2022; Zhang et al., 2022; Yang S. et al., 2020), apoptosis (Liu T. et al., 2022), immune responses, and other physiological processes. These findings align with the changes in the components during black ginseng processing. For example, the secondary saponin Rg5—produced during black ginseng processing—induces apoptosis and autophagy by inhibiting the PI3K-Akt signaling pathway and may become a promising anti-tumor drug against breast cancer (Yang D. et al., 2020; Yang S-H. et al., 2023). Lee et al. (2019) confirmed that black ginseng inhibited the pro-inflammatory mediators iNOS and COX-2 and the pro-inflammatory cytokines IL1β, IL-6, and TNF-α. Protein expression was determined using protein blotting, which confirmed that black ginseng exhibited stronger anti-inflammatory effects compared to fresh ginseng.

The PageRank algorithm was used to calculate the importance of each gene in the protein interaction network. As a result, 10 hub genes were selected for each module, with a total of 70 key genes. Most of these genes are related to tumors and immune and neurological diseases. Some of them may serve as new therapeutic targets, providing innovative ideas for clinical applications.

## 5 Conclusion

This study investigated the changes in the chemical composition and pharmacological effects of black ginseng during processing. The relationship among the compositional changes of ginsenosides (the main active components in ginseng), the substrates of the Maillard reaction (MR; reducing sugars and amino acids), and the melanoidins generated was analyzed. Chromaticity measurements were performed to determine the changes in the color of black ginseng during the reaction. A higher proportion of rare saponins in the system correlates with a lower amino acid content and a higher melanoidin content. High-performance liquid chromatography-mass spectrometry (HPLC-MS) was used to identify the ginsenosides, sugars, and amino acids in the samples and determine the changes in the chemical composition during processing. The goal was to provide a basis for a more comprehensive study of the chemical composition during black ginseng processing and melanoidin formation.

In addition, differential gene analysis and GO and KEGG enrichment analyses of cells exposed to fresh ginseng and black ginseng extracts were conducted using gene chip technology and transcriptomics. We found that black ginseng regulates the proliferation and differentiation of immune-related cells and positively regulates cell adhesion, suggesting broader prospects in treating neurological diseases and tumors as well as immunotherapy. This study is a preliminary exploration of the differences between the pharmacological mechanisms of black ginseng and fresh ginseng, aiming to provide data for in-depth research into their potential mechanisms.

## Data Availability

The original contributions presented in the study are included in the article/supplementary material, further inquiries can be directed to the corresponding author.
